# Einthoven dissertation prizes 2015

**DOI:** 10.1007/s12471-016-0835-5

**Published:** 2016-04-14

**Authors:** E. E. van der Wall

**Affiliations:** Netherlands Society of Cardiology/Holland Heart House, Moreelsepark 1, 3511 EP Utrecht, The Nederlands

For the 27th time in a row the Netherlands Heart Institute (NHI, formerly ICIN) and the Netherlands Society of Cardiology (NVVC) supported the competition for the three best cardiovascular PhD theses, published in the year 2015 [1–5]. The dissertation prize carries the name of one of the greatest Dutchmen in the history of cardiovascular medicine, Willem Einthoven, who in 1902 for the first time recorded the human ECG, for which he received the Nobel Prize in 1924 [6].

This time the jury received a total of 19 PhD dissertations published in 2015. The jury members were very much impressed by the high scientific quality of the PhD fellows. The ultimate selection was based on a combination of several parameters: the curriculum vitae of the candidate, the scientific originality of the PhD thesis and its relevance for the cardiovascular field. In addition, several objective bibliometric parameters were used: 1) the number of articles in first-rate journals both in PubMed and the Web of Science (WOS), 2) the number of citations in WOS, 3) the Hirsch Index, and 4) the contribution as a first author (or shared first author).

Based on a combination of these results, the jury finally selected three nominees: W.L. Verloop (University Medical Center Utrecht), T.P. van de Hoef (Academic Medical Center, Amsterdam), and P.F.A. Teunissen (VUMC, Amsterdam).

The members of the jury were: P.A. Doevendans (Director NHI), D.J. Duncker (Director NHI), J.W. Deckers (Director CVOI), A. Mosterd (Chairman WCN), M.J. Schalij (Chairman Concilium NVVC), and B.J. Mulder (President NVVC).

The three candidates presented their PhD theses at the annual Spring Congress of the NVVC, held at the Congress Centre “De Leeuwenhorst” in Noordwijkerhout, on 31 March and 1 April 2016. We thank the laureates for their excellent presentations and we wish them a prosperous scientific career.

## Summary

### The dreams of renal denervation: a translational approach

In 2009, renal denervation (RDN) was presented for patients with resistant hypertension. The rationale behind RDN is that chronic (hyper)activation of the sympathetic nervous system is the cause of hypertension. However, the exact mechanism behind RDN is unknown. Moreover, it is of interest which patient categories would benefit from RDN, especially since RDN is irreversible, and whether RDN can also be used in other diseases characterised by a (hyper)active sympathetic tone.

In an analysis of all patients referred for RDN, we showed that two-thirds were excluded from treatment: 25 % of these patients appeared to have “white-coat” resistant hypertension and 12 % had a secondary cause of hypertension. This was remarkable since all referred patients had an extensive history of hypertension. We therefore proposed a screening protocol that has been adapted by most Dutch hospitals performing RDN.

To further identify eligible patients, we performed a retrospective analysis to predict successful therapy. We showed a relation between renal function (by means of estimated glomerular filtration rate) and a blood pressure (BP) reduction. To our knowledge, we were the first to observe that patients with chronic kidney disease may benefit from RDN.

We also analysed the effects after RDN in our population. Unlike other centres, we standardised the antihypertensive drug conditions to prevent potential disturbances. We observed a less prominent reduction in BP. Moreover, in a substudy we observed no effect on sympathetic activity despite a BP reduction in most patients. In conclusion, we did not observe significant changes in end-organ damage, such as left ventricular mass.

Besides hypertension, the metabolic syndrome is also characterised by an overactive sympathetic nervous system. Therefore we investigated the effect of RDN on insulin sensitivity, BP, and sympathetic activity in a metabolic population. We showed that RDN did not lead to an improvement of insulin sensitivity at 12-month follow-up, nor did we observe a reduction in sympathetic activity. In this population, we did observe a moderate reduction in ambulatory 24-hour BP. However, this may be explained by the phenomenon of “regression to the mean”, since frequent home-based BP measurements did not change during the 12 months after RDN.

To gain more insight into the RDN mechanism, we designed a porcine model to study the effect on renal vasculature and haemodynamic changes in the kidneys themselves. There was a reduction in microvascular resistance. However, the most prominent observations were a limited penetration up to 2 mm deep in the artery wall and that most of the renal nerves were remote to the vascular lumen and therefore not reached by the delivered radiofrequency energy. Furthermore, we observed a relation between more vascular damage and pronounced haemodynamic effects.

Based on these findings, we investigated whether similar haemodynamic changes could also be observed in hypertensive patients. Directly after RDN, we showed a reduction in the arteriolar resistance. Moreover, we observed that acute changes in arteriolar resistance were significantly associated with the change in BP during follow-up, suggesting this may be a marker for successful RDN.

In conclusion, RDN may be effective to lower BP. However, a large number of patients were non-responders. Our haemodynamic studies may explain these findings.

*W.L. Verloop*

*UMCU*

*Email: W.L.Verloop@umcutrecht.nl*

## Summary

### Novel insights into the complexity of ischaemic heart disease derived from intracoronary pressure and flow velocity measurements

The management of ischaemic heart disease in contemporary clinical practice remains governed by the extent of focal epicardial coronary artery disease. Its diagnosis is principally established by means of invasive coronary angiography, and revascularisation is preferably guided by fractional flow reserve. Despite increasing interest in the functional relevance of epicardial stenosis and its beneficial effect on patient outcomes in the setting of stable ischaemic heart disease, it is important to realise that ischaemic heart disease goes beyond the domain of the coronary circulation that can be assessed by angiography and fractional flow reserve. Both only aim to identify (functionally) important epicardial coronary artery disease, and provide limited or no information on the presence of diffuse epicardial and microvascular disease. These conditions can be identified by the additional assessment of coronary flow parameters, such as coronary flow reserve and microvascular resistance index. We documented that coronary flow reserve has a dominant role over fractional flow reserve in the prognosis of stable ischaemic heart disease after deferral of revascularisation in intermediate coronary stenosis: as long as the coronary flow reserve remains above normal thresholds, the occurrence of a pressure drop along the coronary artery has little effect on clinical outcomes, whereas reduced flow reserve is associated with severely impaired clinical outcomes even in the absence of pressure loss along the coronary artery, suggestive of dominant microvascular disease. These data suggest that the coronary microcirculation, which remains elusive to the interventionalist in contemporary angiography and fractional flow reserve-guided practice, plays a dominant role in the diagnosis and risk stratification in stable ischaemic heart disease patients. This is supported by the observation that the identification of ischaemia-inducing coronary stenosis by fractional flow reserve is influenced by the functional status of the coronary microcirculation, where, for a given stenosis, its ischaemic potential increases with increasing microvascular resistance to coronary blood flow. These observations on the relevance of microcirculatory function and coronary flow for the diagnosis and prognosis of stable ischaemic heart disease have led to the hypothesis that a primary approach towards its diagnosis should be found in the coronary flow characteristics of the vasculature under investigation, instead of the contemporary coronary pressure-guided approach. For this purpose, and since coronary flow reserve has distinct limitations due to its dependence on resting haemodynamics, we described a novel concept termed coronary flow capacity. This approach is governed by the assumption that myocardial ischaemia is unlikely when either coronary flow reserve or maximal flow is above normal thresholds. With increasing impairment of both of these parameters, the likelihood of myocardial ischaemia and impaired clinical outcome increases. Coronary flow capacity indeed allowed more robust risk stratification compared with coronary flow capacity in patients with ischaemic heart disease. Since such a flow-based concept is applicable to all modalities that measure flow, an approach can be anticipated that first determines the severity of ischaemic heart disease by determining the overall flow impairment throughout the myocardium before the epicardial and microvascular contribution to flow impairment are evaluated to determine the dominant origin of flow impairment, and eligibility for coronary revascularisation.

In conclusion, this thesis dives into the complexity of ischaemic heart disease that derives from the complex multi-level involvement of the coronary circulation, including not only focal epicardial, but also diffuse and microvascular abnormalities that impair myocardial perfusion, and proposes a novel flow-based approach to the diagnosis and treatment of ischaemic heart disease patients.

*T.P. van de Hoef*

*AMC Heart Centre, Academic Medical Center, Amsterdam, the Netherlands*

*Email: t.p.vandehoef@amc.uva.nl*

## Summary

### Ischaemic heart disease: from perfusion deficits after microvascular injury to perfusion restoration by arteriogenesis

The prognosis of patients suffering from ischaemic heart disease has rapidly and greatly improved over the course of some 35 years. Microvascular injury, however, remains a threat to the functional recovery of ST-segment elevation myocardial infarction (STEMI) patients. Despite successful treatment with primary percutaneous coronary intervention (PCI), between 40 and 50 % of STEMI patients develop microvascular injury, which is associated with negative remodelling and left ventricular dysfunction, leading to decreased long-term survival, increased morbidity and reduced quality of life.

In this thesis, intracoronary physiology indices were shown to be valuable in predicting the occurrence of microvascular injury and microvascular perfusion deficits after angiographically successful primary PCI. In a prospective clinical study of 60 patients presenting with STEMI, hyperaemic microvascular resistance, measured immediately following angiographically successful PCI, was shown to predict microvascular injury as assessed by cardiac magnetic resonance (CMR) and reduced myocardial blood flow as quantified by positron emission tomography. Furthermore, a cut-off for hyperaemic microvascular resistance (2.5 mmHg/cm · s) was established for predicting extensive microvascular injury. Moreover, in STEMI patients a disbalance was shown between “a disintegrin-like and metalloprotease with thrombospondin type motif no. 13” (ADAMTS13) and von Willebrand factor towards a hypercoagulable sate. Recombinant ADAMTS13 was tested as a therapeutic strategy in myocardial ischaemia-reperfusion injury in a preclinical porcine model. In a translational approach, combining data from our clinical study with preclinical data, we investigated the histopathological correlate of CMR findings of microvascular injury. Areas of CMR-defined microvascular obstruction after acute myocardial infarction were shown to actually represent microvascular destruction and intramyocardial haemorrhage.

In chronic myocardial ischaemia, collateral artery formation or arteriogenesis is potentially a tissue and lifesaving mechanism. Therapeutic strategies aiming to enhance arteriogenesis could be a valuable addition to the therapeutic arsenal of cardiologists treating patients with advanced ischaemic cardiomyopathy. Due to the important side effects of strategies aimed at accelerating arteriogenesis investigated hitherto, such as acceleration of atherosclerosis, to date no such strategy has found its way to clinical practice. The second part of this thesis discusses the coronary collateral circulation in experimental models and humans as well as clinical parameters associated with collateral development in patients. Finally, an elaborate experimental approach to stimulate arteriogenesis was explored. We demonstrate that blocking the interferon alpha and beta receptor using monoclonal antibodies accelerates arteriogenesis in mice without influencing atherosclerosis.

In conclusion, this thesis provides new and important insights regarding vascular damage and healing in cardiac ischaemic syndromes which will ultimately support the design of therapeutic strategies to combat microvascular injury and to stimulate collateral artery growth.

*P.F.A. Teunissen*

*Interventional Cardiology VU University Medical Center, Amsterdam, the Netherlands*

*Email: pf.teunissen@vumc.nl*

Dr. van de Hoef won the first prize, dr. Verloop the second prize, and dr. Teunissen the third prize.

## References

[1] van Gilst WH, van der Wall EE (2010). Einthoven dissertation prize 2010. Neth Heart J.

[2] van der Wall EE, Schalij MJ, Umans V, van Gilst WH (2012). Einthoven dissertation prizes 2011. Neth Heart J.

[3] van der Wall EE, van Gilst WH, Schalij MJ, Umans V (2013). Einthoven dissertation prizes 2012. Neth Heart J.

[4] van der Wall EE, Umans VA (2014). Einthoven dissertation prizes 2013. Neth Heart J.

[5] van der Wall EE, Deckers JW, Umans VA (2015). Einthoven dissertation prizes 2014. Neth Heart J.

[6] Umans VA, van der Wall EE (2014). 80 years of Netherlands Society of Cardiology. Neth Heart J.

